# Psychiatric Admissions in NHS Lothian – What Can We Do Better?

**DOI:** 10.1192/bjo.2024.495

**Published:** 2024-08-01

**Authors:** Ruairidh Allen, Konal Dissanayake, Milly Green, Kenneth Murphy, Douglas Murdie

**Affiliations:** 1 University of Edinburgh Medical School, Edinburgh, United Kingdom; 2 NHS Lothian, Edinburgh, United Kingdom

## Abstract

**Aims:**

Psychiatric services are under increasing pressure to provide effective patient care with diminishing resources. In NHS Lothian, there is a sector-based model and chronic issues with lack of inpatient beds.

We aim to examine the admission to discharge process at the Royal Edinburgh Hospital (REH) for patients within the North-West sector of Edinburgh (NW) to identify areas for improvement.

**Methods:**

We collected data for NW admissions and discharges from the five General Adult Psychiatry wards in REH in 2023, two of which are allocated NW wards.

Admissions to the Intensive Psychiatric Care Unit were excluded as they indicated differing severity, and discharges via the ‘long-stay’ ward were excluded due to other factors delaying discharge.

Data was collected from NHS Lothian Analytical Services and anonymised in line with NHS Information Governance Policy.

Qualitative data was collected anonymously from staff within NHS Lothian in the form of an online questionnaire to identify strengths and weaknesses of the current processes.

**Results:**

In 2023 there were 133 discharges of NW patients in REH. The average age was 39 years old and most common diagnosis was a psychotic illness (36%).

Qualitative data identified that admitting patients to hospital is increasingly challenging due to capacity issues and the lack of a community transfer plan.

53% of NW patients were admitted to NW wards. 27% of patients were moved between wards during their admission.

Length of stay (LOS) and readmission rates were used as proxy measures to examine patient outcomes. Patients who remained on the same ward during their admission had an average LOS of 28 days. 22% were re-admitted within the calendar year. Outcomes were no better when patients remained on their sector ward.

Patients who moved ward during their admission to hospital had an average LOS of 47 days. 45% were re-admitted.

**Conclusion:**

A lack of bed capacity is having a negative impact on patient care in NHS Lothian. Staff expressed concerns about the admission process and patients are moving wards during acute episodes of care to accommodate a sector-based model and chronic lack of capacity. Lack of continuity during admissions may be contributing to longer admissions and more re-admissions, further impacting on capacity.

A review of the strategic planning of NHS Lothian Psychiatric care is required, including capacity planning, admission protocols and policies on boarding patients. We will disseminate these results to support this process and any future work into this topic.

